# *Sarcoptes scabiei* infestation does not alter the stability of ectoparasite communities

**DOI:** 10.1186/s13071-016-1659-2

**Published:** 2016-07-01

**Authors:** João Carvalho, Emmanuel Serrano, Nathalie Pettorelli, José E. Granados, Miguel A. Habela, Sonia Olmeda, Carlos Fonseca, Jesús M. Pérez

**Affiliations:** Department of Biology & CESAM, University of Aveiro, 3810-193 Aveiro, Portugal; Servei d’Ecopatologia de Fauna Salvatge (SEFaS), Departament de Medicina i Cirurgia Animals, Universitat Autònoma de Barcelona, E-08193 Bellaterra, Barcelona, Spain; Institute of Zoology, Zoological Society of London, Regent’s Park, London, NW1 4RY UK; Espacio Natural de Sierra Nevada, Carretera Antigua de Sierra Nevada, km 7, E-18071, Pinos Genil, Granada, Spain; Parasitology & Parasitic Diseases Animal Health Department, Veterinary Faculty, University of Extremadura Av. Universidad, s.n., E-10003 Cáceres, Spain; Departamento de Sanidad Animal, Universidad Complutense de Madrid, Av. Puerta de Hierro, s.n., E-28040 Madrid, Spain; Departamento de Biología Animal, Biología Vegetal y Ecología, Universidad de Jaén, Campus Las Lagunillas, s.n., E-23071 Jaén, Spain

**Keywords:** *Capra pyrenaica*, Community ecology, Ecosystem engineer, Multiparasitism, *Sarcoptes scabiei*

## Abstract

**Background:**

The host represents a heterogeneous ecosystem where multiple parasite species co-occur and interact with each other for space and resources. Although these interactions may rule the features of an infracommunity and may shape the infracommunity response to external perturbations, the resilience of ectoparasite communities to new infestations remains poorly explored.

**Methods:**

We analysed the composition of the ectoparasite communities found on 214 individual Iberian ibexes (*Capra pyrenaica*) inhabiting the Sierra Nevada Natural Space, southern Spain. Using classification and regression trees, we explored how the presence of *Sarcoptes scabiei* (a highly contagious mite), the off-host environment and the host sex govern the prevalence and abundance of lice and ticks. Null model analysis was applied to assess the impact of *S. scabiei* on the structure of the ectoparasite communities.

**Results:**

Our results suggest that *S. scabiei* infestation acts in tandem with off-host environment and host sex to define the prevalence and abundance of lice and ticks. We also provided evidence for differences in species co-occurrence only at the early stages of *S. scabiei* infestation. Regarding species diversity, we recorded that ectoparasite communities in scabietic ibexes reached a high richness faster than those in healthy individuals.

**Conclusions:**

Even though we show that ectoparasite burden is correlated with *S. scabiei* infestation, off-host environment and host sex, the species response to *S. scabiei* infestation and climate seem to be highly variable and influenced by ectoparasite life-history traits. Ectoparasite communities also appear resilient to perturbations which is in agreement with what was previously reported for endoparasites. Future refinement of sample collection and the incorporation of ecological and epidemiological-related variables may allow us to establish causal effects and deepen the knowledge about the mechanisms and consequences of ectoparasite interactions.

## Background

The host represents a heterogeneous ecosystem encompassing a wide range of linked microhabitats (Table 1). From the host’s skin surface to the host’s inner organs, each microhabitat has particular characteristics that influence the prevalence and abundance of parasite populations [1]. However, the tissue tropism and the fundamental niche of a parasite species are not only influenced by microhabitat suitability. The host immune and defensive responses (top-down regulation) as well as the within-host competition (exploitation, apparent and interference) for space and resources (bottom-up regulation) are believed to act, synergistically or not, in the maintenance of parasite specificity and parasite burdens [2]. Gaining a broader understanding of parasite interactions within host is of paramount importance not only because such interactions drive several features of an infracommunity but also because may shape disease dynamics [3].

Although the vast majority of studies reporting interspecific interactions has been achieved at laboratory level and focused on helminths and microparasites [4], recent experiments have started to explore the interactions between endo and ectoparasites [5, 6]. However, thus far the mechanisms of parasite co-infection and the resilience of infracommunities to external perturbations remain poorly understood [7, 8]. For instance, infracommunity interactions between different ectoparasite taxa have only recently begun to receive attention. By inducing external perturbations through the application of acaricides in the ectoparasite community of a wild mammal, recent research showed that ectoparasites interactions affect host susceptibility to other ectoparasites as well as the distribution of ectoparasites among hosts [9, 10]. It also suggested that species with differing life-history traits are affected by perturbations in different ways [9, 10]. Yet how ectoparasite communities respond to infestations caused by highly contagious mites, namely those infestations able to alter hosts’ phenotype, remains unclear. Several parasite species may be involved, directly or indirectly, in ecosystem engineering processes either causing immune and structural changes in their hosts or altering host’s traits [11]. Parasite-mediated changes can range from antagonistic to facilitating the establishment and growth of existing or subsequent parasites [12]. Consequently, the infestation by one virulent parasite may have important implications in the dynamics of within-host ectoparasite communities.

**Fig. 1:**
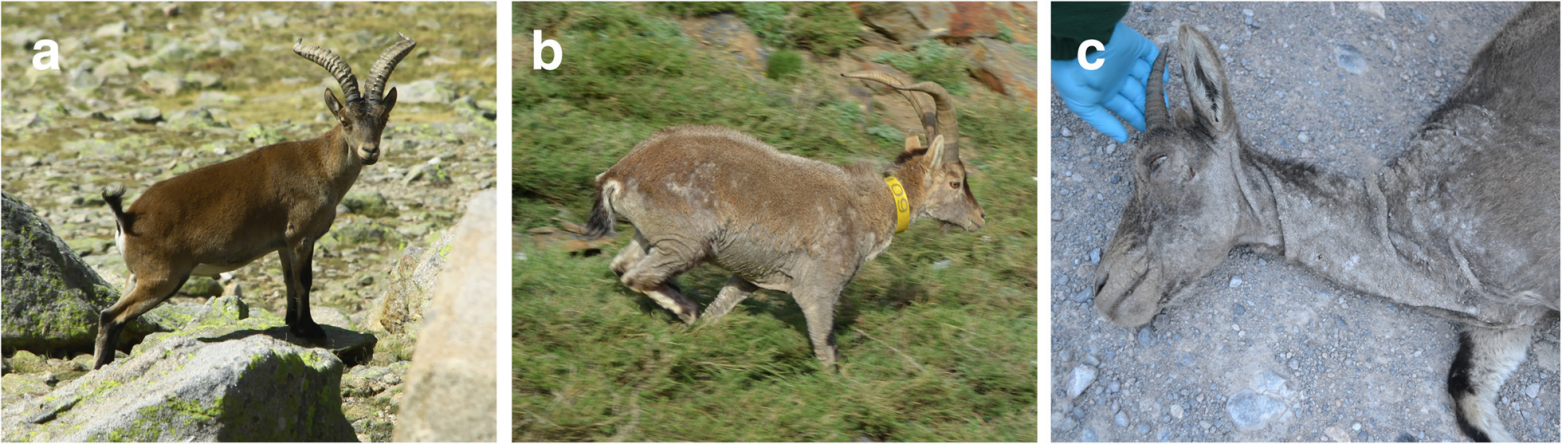
Wild ibexes exhibiting different stages of mange severity. **a** Healthy ibex with no evidence of skin lesions (continuous microhabitat). **b** Mildly infested ibex presenting extensive alopecia on the face, abdomen, elbow and knees (loss of microhabitat). No evidence for hyperkeratotic and parakeratotic lesions. **c** Advanced case of *S. scabiei* infestation accompanied by an almost complete alopecia on the face, ears and neck (microhabitat becomes divided into several patches). Note thick crusts on the muzzle which may indicate the deficiency of a hypersensitivity response

*Sarcoptes scabiei* has become endemic in many mammal populations across Europe, affecting in particular the population dynamics of wild ungulates [13]. Dramatic structural damages and functional changes in the host skin can result from mange infestation (Fig. 1). These lesions are particularly evident within the winter/spring period [14, 15]. The lesions are first localised but after several weeks the pruritic skin is accompanied by erythematous eruptions, hyperkeratosis, alopecia and hypersensitivity [16]. By altering this particular microhabitat, *S. scabiei* may play an important role as an ecosystem engineer. This study aims to test a series of hypotheses regarding how engineering parasites such as *S. scabiei*, off-host environment and host sex rule the prevalence, abundance and structure of the remaining ectoparasite (in this case lice and ticks) communities, using the Iberian ibex (*Capra pyrenaica*) as a model host. Lice are small, wingless and flattened ectoparasites, living permanently on their hosts. One group (Ischnocera: Trichodectidae, represented in our study by *Bovicola crassipes*) feeds on host skin debris, and the other one (Anoplura: Linognathidae, represented in our study by *Linognathus stenopsis*) has a hematophagous behaviour. These groups are endowed with specific adaptations enabling them to live clinging to fur. In contrast to lice, ticks are long-lived ectoparasites, spending long intervals off the host between blood meals [17]. Given this contrast in life-history traits, we expect that lice burdens might be particularly influenced by *S. scabiei* infestation and tick burdens might be mainly sensitive to off-host environment (Hypothesis 1a). As the prevalence and abundance of both groups vary seasonally (lice, see [18]; ticks, see [19]), we also expect a pronounced seasonal pattern in ectoparasite (lice and ticks) burdens (Hypothesis 1b). Gender-biased parasitism in vertebrates has often been reported [20]. Although exceptions do exist [21], male vertebrates are generally more parasitised than females due to their lower immunocompetence [22], higher mobility [23] and larger body size [24]. Because ibexes are dimorphic [25], we expect that host sex will interact with *S. scabiei* infestation and off-host environment in shaping the prevalence and abundance of lice and ticks (Hypothesis 2). Parasite communities may be structured through direct and/or indirect interactions between multiple parasite species [26]. Parasites that alter particular traits of their hosts, creating or modifying existing microhabitats, constitute a noteworthy example of how a parasite species may promote or suppress the establishment of subsequent parasites and/or disrupt the structure of native parasite communities [1]. As *S. scabiei* causes physical damages in the host skin and coat, we hypothesised that *S. scabiei* may promote changes in the structure and composition of ectoparasite (lice and ticks) communities (Hypothesis 3).

## Methods

### Ethics statement

This study complies with the Spanish and the Andalusian laws regarding bioethics and animal welfare. The Sierra Nevada National Park approved this study.

### Study area

The Sierra Nevada Natural Space (SNNS; Latitude 36°00'–37°10'N, Longitude 2°34'–3°40'W) holds the largest and best known population of Iberian ibex in Andalusia [27]. This area is characterised by a Mediterranean subarctic climate experiencing seasonal and altitudinal gradients of temperature and precipitation [28]. The average monthly temperature ranges from -5 °C in February to 17 °C in July and may vary between 12 and 16 °C below 1,500 m and 0 °C above 3,000 m. Annual average precipitation is approximately 600 mm [29]. The snow is generally present between December and May; vegetation growth mainly occurs during summer months (June–August).

### Sampling procedure

The data were collected from 214 ibexes (131 males and 83 females) shot-harvested between 2002 and 2008 on a monthly basis as part of a sarcoptic mange control program devoted to manage ibex density and the spread of mange in the SNNS. Iberian ibexes of both sexes and all ages were shot-harvested whenever mange lesions were detected. Additionally, some ibexes were selectively harvested in the context of a population management program devoted to ensure the equilibrium of the Iberian ibex population. Sex was assigned by visual inspection while age determination was performed through horn-segment counts [30]. Ibexes were grouped into three age classes: kids (0 ≤ age ≤ 1 year, *n* = 20), yearlings (1 < age ≤ 2 years, *n* = 42) and adults (> 2 years, *n* = 152); kids were excluded from the analysis. The severity of *S. scabiei* infestation was assessed by measuring the surface of scabietic lesions and by digesting five skin fragments (6.25 cm^2^ each [31]). The digestion procedure was carried out overnight using a 5 % potassium hydroxide (KOH) at 40 °C. Each fragment was digested separately. The resulting products were re-suspended and analysed using a stereomicroscope for mite counts. The mean of the five counts was used as a proxy of mite load. The animals were categorised as healthy (ibexes without skin lesions), mildly infested (skin surface affected ≤ 50 %) and severely infested (skin surface affected > 50 %) [31]. Ectoparasites (lice and ticks) were systematically removed and counted by three observers during 15 min. Therefore, the number of lice and ticks represents an abundance index. Lice were treated with lactic acid and mounted with DePex medium. Ticks were fixed in 70 % ethanol. Lice and ticks were identified to the species level using the available descriptions [32–34].

### Environmental variables

Climate and environmental moisture are some of the main factors governing the prevalence and abundance of ectoparasites and determine which species are found in a particular geographical extent. For instance, the temperature rules the duration of ticks’ life-cycle and together with environmental humidity and water availability shape their development and activity [19]. Lice are particularly sensitive to the on-host environment; however their reproductive traits, their survival off the host and their transmission could be increased by favorable off-host temperature and/or humidity [35]. Two variables were considered while assessing the role of off-host environment processes on the prevalence and mean abundance of lice and ticks. The Normalized Difference Vegetation Index (NDVI [36]) was included in the analysis as a proxy of environmental moisture [37]. This variable was previously associated with ectoparasite burdens [35] and was recently linked to the dynamics and consequences of *S. scabiei* infestation in the Iberian ibex population of SNNS [38]. The NDVI was gathered from the MODIS repository (Moderate Resolution Imaging Spectroradiometer; http://modis.gsfc.nasa.gov) at a spatial resolution of 500 m and at a bi-monthly temporal resolution. Monthly mean temperature was acquired from the Mountain Meteorological Services website (http://meteoexploration.com/forecasts/Sierra-NevadaES/). The month and season temperature was calculated as the mean of measures taken at constant (daily) time intervals. The values assignment was held considering the ectoparasites (lice and ticks) life-cycle and those periods when ticks are known to spend long intervals off-host (Table 2). To control for seasonal fluctuations in ectoparasite burdens and host-parasite interactions, we included two indices (the winterness and the springness) derived from the Julian day. Both indices range between -1 and 1. Winterness takes higher values (close to 1) if the Julian day represents the winter and lower values (close to -1) if the Julian day represents the summer. Springness behaves similarly, except that higher values (close to 1) represent the spring (see [39] for an introduction of circular statistics).

**Table 1:** Definition of terms

Ecosystem engineer	Concept coined by [62] that refers to organisms that directly or indirectly alter the environment in which they occur. Their action may modify or create new habitats through their own physical structures (autogenic engineers) or through their activities (allogenic engineers).
Functional response	Differences in tissue preferences or resources used by a species in response to interspecific competition [26]. It is an indicator that different parasite species are interacting.
Infracommunity	Includes all the infrapopulations that colonise a single host or an organ and interact with each other for space and resources [40].
Microhabitat	Small-scale environment where an organism or an assemblage of organisms naturally occur and interact, both directly and indirectly, with the biotic and abiotic elements.
Numerical response	Positive or negative variation of a particular infrapopulation size when facing the presence of another species [26]. It is an indicator that different parasite species are interacting.
Virulence	Effects of an infection in host fitness, i.e. severity of the disease caused by a particular organism (damage-related concept [63]). It could also express the transmissibility of an organism, i.e. its capacity to grow and proliferate within a host [64].

**Table 2:** Variables selected by the models and its respective measure units. In this study, we defined four calendar-based seasons

Group of ectoparasites	Variable (unit)	Abbreviation
Lice	Average temperature for the month of sampling (°C)	Temp
	Average NDVI for the month of sampling (mm)	NDVI
Ticks	Average summer temperature for the year of sampling (°C)	TS_t0
	Average winter temperature for the year of sampling (°C)	TW_t0
	Average autumn temperature for the year of sampling (°C)	TA_t0
	Average spring NDVI for the year of sampling (mm)	NDVIS_t0
	Average autumn NDVI for the year of sampling (mm)	NDVIA_t0
Lice and Ticks	Host sex	Sex
	Degree of *S. scabiei* infestation	Mange
	Springness	Springness
	Winterness	Winterness

### Prevalence and mean abundance of ectoparasite species

The prevalence and mean abundance [40] of ectoparasite (lice and ticks) species were estimated using the software Quantitative Parasitology 3.0 [41]. The confidence interval (CI) for prevalence was calculated through Sterne method [42], while bootstrap (BCa, 2,000 replications) were used to estimate the CI for the mean abundance.

### Classification and regression trees

The contribution of *S. scabiei* infestation, off-host environment and host sex to the prevalence and mean abundance of lice and ticks (numerical response) were explored through classification (prevalence) and regression (abundance) trees (CART [43]). CART’s main advantage relies on its flexibility to handle interactions and nonlinearities among variables, predictive power and easy interpretation. The two main issues in constructing a reliable and informative tree model are to find good data splits and to avoid data over-fitting. The first can be addressed through the determination of information gain or node impurity measures (e.g. entropy, Gini index of diversity or misclassification error) whereas model over-fitting is reduced by pruning the tree [44]. In our analysis, the information gain was applied to select the best split and the complexity parameter was used in order to prune the tree and represent the data as simple and interpretable as possible. The prediction error rate in cross-validation procedure was used to assess the model reliability. CART models were fitted using the ‘*rpart*’ library [45] and plotted using the ‘*rpart.plot*’ library [46], R statistical software version 3.1.3 [47].

### Null models

Null model analysis was used to explore whether pairwise associations among ectoparasite (lice and ticks) species occurred by chance or were influenced by the severity of *S. scabiei* infestation (functional response); these were also used to study the patterns of species diversity (richness) among healthy and scabietic hosts.

To assess patterns in species co-occurrence, three datasets, each representing a degree of *S. scabiei* infestation, were organised as r × c matrices where each row represents a species of ectoparasite and each column an individual ibex. The cells in the matrix denote the presence (1) or the absence (0) of a particular species of ectoparasite in a particular ibex. We assume individual ibexes as replicates to unveil repeated patterns of species co-occurrence. Co-occurrence was assessed by computing the number of checkerboard species pairs, the default co-occurrence index (C-score) and the number of species combinations. The number of checkerboard species was originally defined as an indicator of species competition [48]. This statistic identifies the number of pairs that do not occur together in any site, i*.*e. two or more ectoparasite species (lice and ticks) have mutually exclusive distributions among the sampled ibexes. The C-score is a measure of “checkerboardedness” that expands the concept of “checkerboard distributions”. C-score quantifies the average number of checkerboard "units" for each species pair [49]. In our study, C-score determines the randomness of the distribution of *n* ectoparasite species (lice and ticks) through the sampled ibexes. Higher values of C-score are associated to a more segregated matrix/distribution, whereas a lower C-score is associated to a more aggregated matrix/distribution. The number of species combinations calculates the number of unique combinations represented in each ibex. In a competitively structured community, the following assumptions must be fulfilled: (i) the C-score should be significantly greater than expected by chance (O > E), and/or (ii) the number of checkerboard pairs of species should be larger than expected by chance (O > E), and/or (iii) the number of species combinations should be lower than expected by chance (O < E). In our analysis, we used a fixed-equiprobable algorithm in which we kept the observed rows fixed, i.e. the number of occurrences of each species in the null communities and in the original dataset is the same, and the columns were treated as equally likely, i.e. all ibexes share the same suitability to be invaded by an ectoparasite. The indices were calculated for each presence/absence matrix and compared with the expected indices computed for 5,000 randomised communities through Monte Carlo procedures.

Species richness is the most straightforward and easy-to-interpret measure of species diversity. In this study, one single dataset was created to develop species accumulation curves for each stage of *S. scabiei* infestation. The dataset was organised as a matrix where each row represents an ectoparasite species and each column the abundance of ectoparasites by stage of *S. scabiei* infestation. Species richness was assessed through a species accumulation curve in which the number of parasite species is measured as a function of sampling effort, e.g. the number of parasite individuals identified [50]. The shape and slope of the curve towards its asymptote are influenced by the prevalence and abundance of each parasite species and may vary considerably between infracommunities. The analyses were performed using the EcoSim 7.72 software [51].

## Results

Among the ibexes analysed, 134 individuals (56 females and 78 males) were infested by *S. scabiei*. Apart from this mite, we identified two lice species (*Bovicola crassipes* and *Linognathus stenopsis*) and six species of ticks (*Dermacentor marginatus*, *Haemaphysalis punctata*, *Haemaphysalis sulcata*, *Ixodes ricinus*, *Rhipicephalus bursa* and *Rhipicephalus turanicus*). Prevalence and mean abundance were estimated for each ectoparasite species (Table 3). *Ixodes ricinus* was not considered in our analysis due to the small number of individuals collected (*n* = 2).

**Table 3:** Prevalence and mean abundance of ectoparasite (lice and ticks) species collected from 194 Iberian ibexes (yearling and adults) of both sexes. The confidence interval (CI 95 %) for prevalence and mean abundance are also presented

Species	Period	Total no. examined	No. infested	Prevalence (95 % CI)	Mean abundance (95 % CI)
*Bovicola crassipes*	Annual	194	43	0.22 (0.17–0.29)	2.19 (1.13–5.56)
	Winter	80	22	0.28 (0.19–0.39)	3.38 (1.25–10.21)
	Spring	72	15	0.21 (0.13–0.32)	1.76 (0.69–5.06)
	Summer	11	3	0.27 (0.07–0.59)	1.09 (0.09–3.45)
	Autumn	31	3	0.09 (0.01–0.25)	0.48 (0.03–1.94)
*Linognathus stenopsis*	Annual	194	39	0.20 (0.15–0.27)	3.49 (1.57–8.23)
	Winter	80	23	0.29 (0.20–0.40)	5.18 (1.92–17.24)
	Spring	72	9	0.13 (0.06–0.22)	2.72 (0.44–11.43)
	Summer	11	2	0.18 (0.03–0.50)	2.64 (0.00–9.45)
	Autumn	31	5	0.16 (0.07–0.34)	1.26 (0.35–3.32)
*Dermacentor marginatus*	Annual	194	10	0.05 (0.03–0.09)	0.21 (0.08–0.52)
	Winter	80	5	0.06 (0.03–0.14)	0.34 (0.08–1.24)
	Spring	72	5	0.07 (0.03–0.15)	0.18 (0.04–0.47)
	Summer	11	0	na	na
	Autumn	31	0	na	na
*Haemaphysalis* sp.	Annual	194	34	0.18 (0.13–0.22)	0.64 (0.41–0.99)
	Winter	80	21	0.26 (0.17–0.37)	0.95 (0.56–1.79)
	Spring	72	6	0.08 (0.03–0.20)	0.40 (0.13–1.11)
	Summer	11	1	na	na
	Autumn	31	6	0.19 (0.09–0.37)	0.52 (0.19–0.97)
*Rhipicephalus* sp.	Annual	194	92	0.47 (0.40–0.55)	3.38 (2.64–4.30)
	Winter	80	41	0.51 (0.40–0.62)	3.40 (2.38–4.80)
	Spring	72	35	0.49 (0.37–0.60)	3.94 (2.57–5.56)
	Summer	11	5	0.46 (0.20–0.74)	4.82 (1.09–14.82)
	Autumn	31	11	0.36 (0.21–0.53)	1.48 (0.68–2.71)

**Fig. 2:**
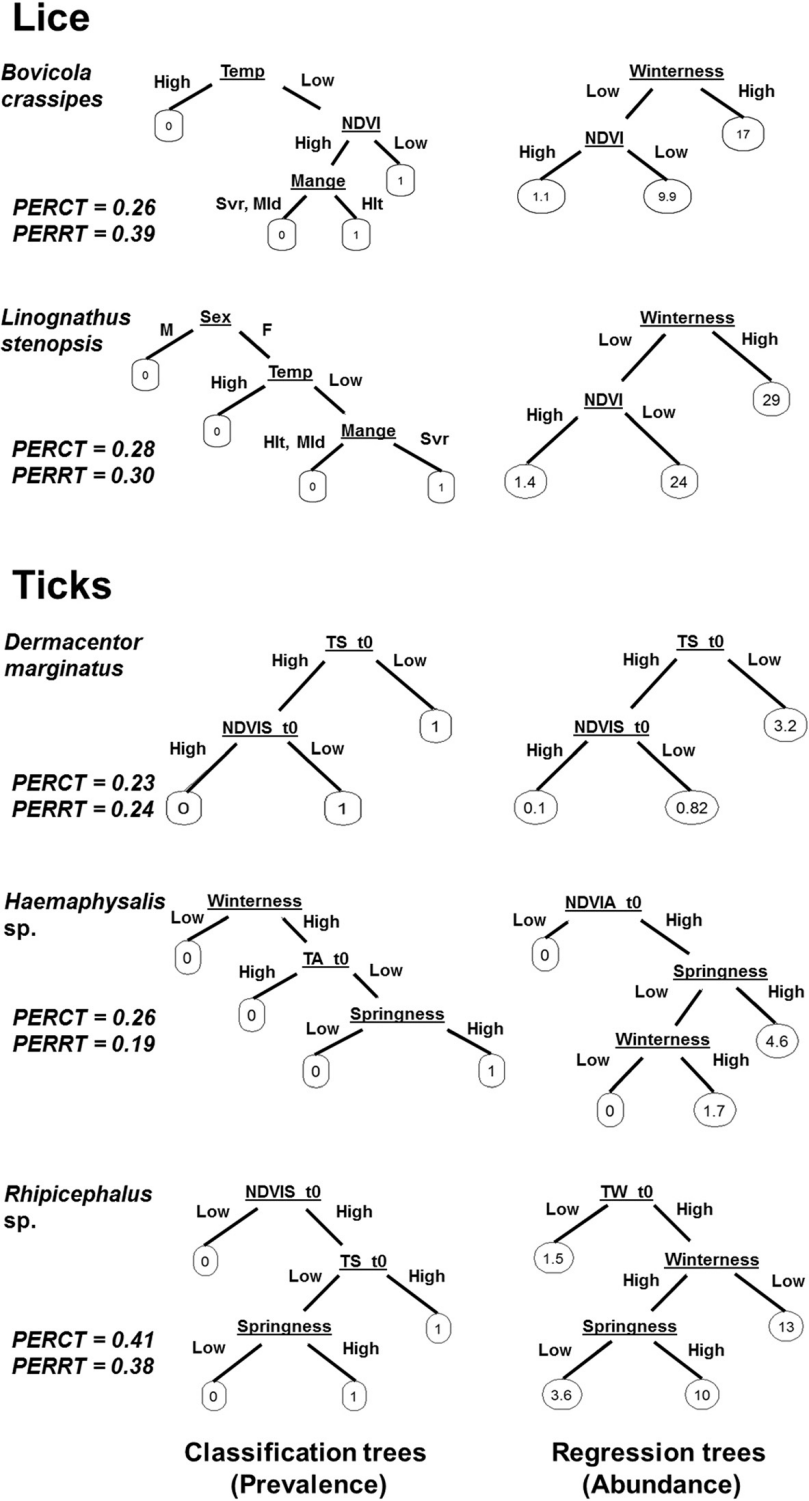
Classification (left) and regression (right) trees from analyses to assessing the influence of *S. scabiei* infestation, environment and host sex on the prevalence and abundance of lice and ticks. Each node represented by the variable abbreviation (variable names as shown in Table 2) defines a split of the dataset and the branches define the path taken by each observation. Ellipses represent the terminal nodes of the tree model. The terminal nodes of classification tree models (left) indicate if an ectoparasite species is present (1) or absent (0) in relation to *S. scabiei* infestation, environment and host sex. The terminal nodes of regression tree models (right) express the variations in ectoparasite abundance in relation to the aforementioned variables. The prediction error rate for each model is presented in bold. *Abbreviations*: PERCT, prediction error rate for classification trees; PERRT, prediction error rate for regression trees

Classification tree results corroborate our first hypothesis (Hypothesis 1a) with *S. scabiei* only influencing the prevalence of lice species. However, *S. scabiei* affected lice species in different ways (Fig. 2). The presence of *S. scabiei* had a negative influence on the prevalence of *B. crassipes* but favored the prevalence of *L. stenopsis.* As expected (Hypothesis 1b), we detected a pronounced seasonal pattern in lice and ticks infestation. Abundances of lice and ticks increased up to a peak in winter/spring and then decreased during summer, which coincided with a period of relatively high temperature and low environmental humidity (Table 3). The influence of off-host environment on the prevalence and abundance of ectoparasites was evident in lice and ticks (Fig. 2).

Our classification and regression tree analyses indicated that host sex may interact with *S. scabiei* infestation and environmental drivers in shaping the prevalence and abundance of one louse species, *L. stenopsis*, which mainly occurred in infested female hosts. We did not, however, detect a clear influence of the host sex on tick burdens (Fig. 2).

**Fig. 3:**
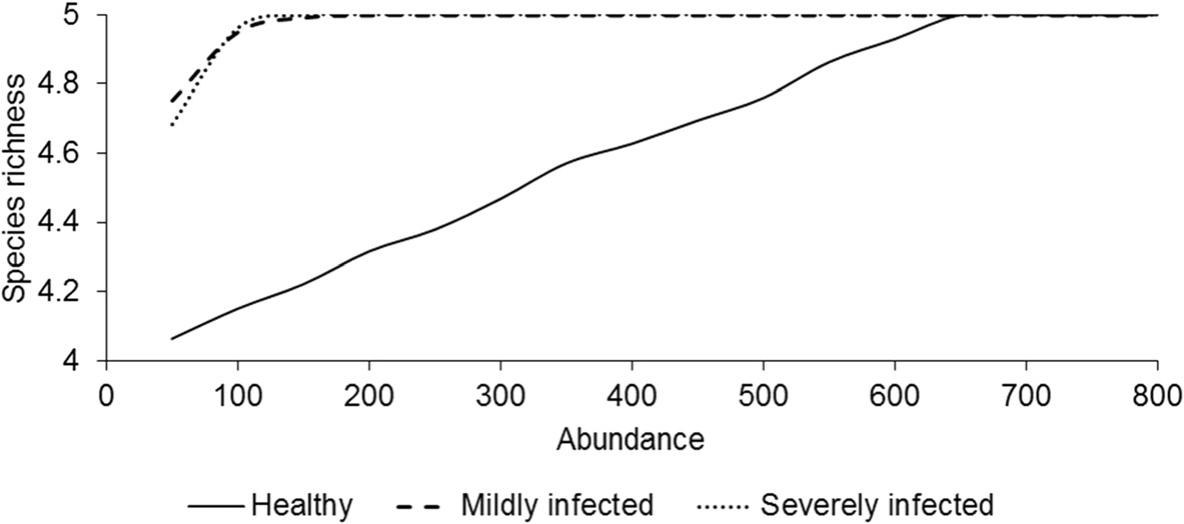
Species accumulation curves showing the rates at which new parasite species are found (species richness) as a function of sampling effort measured as the total number of parasites (abundance) in healthy, mildly infested and severely infested hosts

Interestingly, the diversity of ectoparasites was observed to peak steeper in mildly- and severely infested animals than in healthy individuals (Fig. 3). Furthermore, the null model results suggest that the presence of *S. scabiei* altered the structure of the infracommunities only at the early stages of infestation. Co-occurrence analyses indeed showed that the observed C-score was only higher than expected by chance (O > E) for mildly infested animals (*P*-value < 0.05). This result suggests that a more segregated ectoparasite (lice and ticks) distribution occurred among the ibexes we sampled. However, no statistical significance for the C-score was detected for healthy and severely infested ibexes (*P*-value > 0.05), which suggests that there is no significant evidence for competitive exclusion in these groups. The observed number of checkerboard species combinations was not significantly lower or higher than expected. There was no reason to reject the null hypothesis that the number of checkerboard pairs was random, which means that no mutually exclusive distributions of lice and ticks were detected among the sampled ibexes. The same results were achieved for the number of species combinations suggesting that species co-occurrences were random at mildly and severe stages of *S. scabiei* infestation (Table 4).

**Table 4:** Co-occurrence analysis considering healthy ibexes and two stages of *S. scabiei* infestation/mange severity (mildly and severely infested hosts). The significance between observed (O) and expected by chance (E) values of the C-score, number of checkerboards and number of species combinations is presented for presence/absence matrices of ectoparasite communities of 214 Iberian ibexes

Index	Mange severity	Observed Index	Mean of simulated indices	Variance of simulated indices	*P-*value (O ≤ E)	*P-*value (O ≥ E)
C-score	Healthy	89.00	92.05	5.21	0.07	0.93
	Mild	80.50	74.86	3.60	0.99	0.02
	Severe	50.30	52.21	3.11	0.14	0.87
Number of checkerboards	Healthy	2.00	2.57	1.07	0.40	0.78
	Mild	1.00	1.28	0.76	0.63	0.82
	Severe	1.00	0.75	0.48	0.86	0.60
Number of species combinations	Healthy	13.00	13.82	0.93	0.33	0.91
	Mild	16.00	15.66	1.16	0.79	0.57
	Severe	15.00	14.92	1.11	0.70	0.66

## Discussion

Co-occurring ectoparasite species interact with each other and with on-host and off-host environment. Despite such interactions may govern all features of an infracommunity, their synergistic or antagonistic effects have seldom been assessed. We suggest that the combination of on-host and off-host features is necessary to understand the dynamics of ectoparasite communities in the wild.

**Fig. 4:**
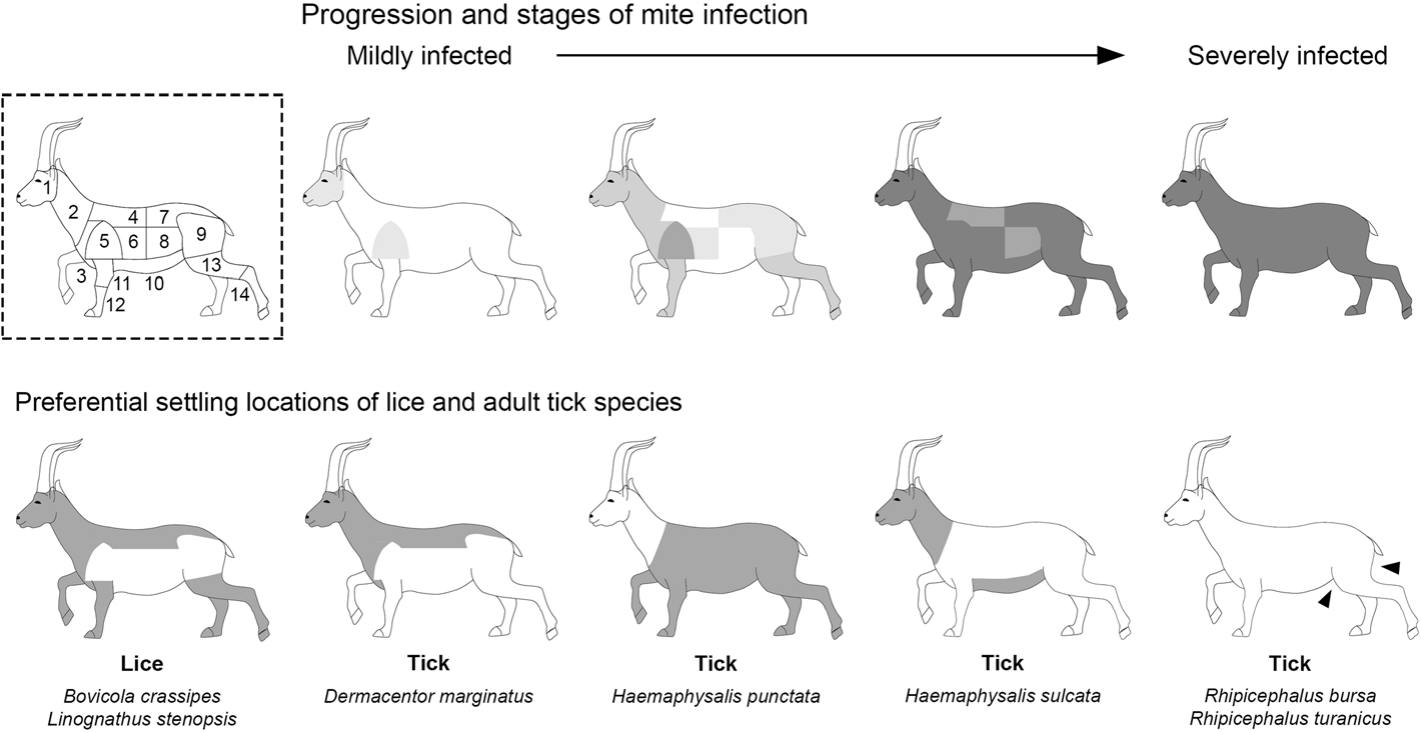
Schematic representation of the preferential use of specific microhabitat areas (shaded in grey) by lice and ticks, and the spread of *S. scabiei* infestation in ibexes. The divergence in space use between lice, ticks and *S. scabiei* and the existence of unoccupied microhabitat patches even during the *S. scabiei* infestation are some reasons to explain the high prevalence and abundance of particular species in scabietic ibexes. *Key*: 1, face; 2, neck; 3, chest; 4, back; 5, shoulder; 6, costal area; 7, lumbar sacra; 8, flanks; 9, pelvis; 10, abdomen; 11, elbow; 12, carpus; 13, groin; 14, tarsus. Different shades of grey indicate a gradient of intensity of mite infestation, light (mild infestation) to dark (severe infestation). The spread of *S. scabiei* and the areas of the body of Iberian ibex follow partially the division of [15]

A distinctive feature of our study showed that structural changes in the host skin and coat caused by allogenic engineers such *S. scabiei* may act in tandem with other factors in defining the prevalence and abundance of ectoparasites, namely lice species. We found however that the impact of *S. scabiei* infestation on the ectoparasite community varies between species with differing life-history traits. For instance, whereas *B. crassipes* was more prevalent in healthy ibexes, *L. stenopsis* was particularly prevalent in severely infested hosts. Although our sampling effort and data collection protocols did not allow us to interpret these variations with confidence, we propose two possible explanations, which are not mutually exclusive. The establishment of *S. scabiei* modifies the on-host environment and decreases the microhabitat suitability to other ectoparasites. However, the divergence in the use of host resources by lice and ticks (the principle of limiting similarity, see [52]) and the existence of several unoccupied niches (Fig. 4) may explain why *S. scabiei* infestation has no adverse effects on the prevalence and abundance of certain ectoparasite species. Additionally, *S. scabiei* debilitates the host and induces changes in the immune response which generally increases host susceptibility to parasitism. Note that large ectoparasitic infestations generally reflect an immunocompromised state of the host individuals [53]. We further suggest that ectoparasite life-history traits provide plausible explanations for the diversity of responses to *S. scabiei* infestation. *Bovicola crassipes* (chewing louse) is indeed perfectly adapted to move within the fur which makes it particularly vulnerable to microhabitat loss. Alopecia would increase the susceptibility of this species to off-host environmental variability, therefore reducing lice survival and burdens in scabietic ibexes. In turn, *L. stenopsis* (sucking louse) is endowed with modified mouthparts for piercing host skin. This adaptation is likely to make this species less prone to microhabitat loss.

Beyond the effects of *S. scabiei* infestation, our results suggest that the prevalence and abundance of lice and ticks follow a pronounced seasonal pattern and vary with off-host environment and host sex. We recorded slightly higher abundances of lice at colder and dryer periods. This trend was previously reported in lice populations occurring in sheep [54], feral ponies [55] and donkeys [18]. For ticks, we reported that *D. marginatus* and *Haemaphysalis* sp. were more prevalent and abundant under average low temperatures, which coincided with the winter/early spring periods. The genus *Rhipicephalus* recorded the highest prevalence and abundance among the species identified. The increasing activity of this genus seems to be correlated with humid and warm winter/spring seasons which contrasts with the results reported in other studies (northern Iran [56]; northern Greece [57]). We believe that host senility and immunocompromised state caused by *S. scabiei* infestation coupled with diet impoverishment noticeable in the winter contribute to higher burdens of parasitism during this period. Collectively, our results corroborate empirical evidence about the role of climate in the regulation of ticks’ prevalence and abundance. However, we realise that each tick species has its own phenology which poses complications for comparative studies and for disentangling the effects of life-history traits, *S. scabiei* infestation and environmental conditions on the features of ticks’ communities.

We reported a weak but female-biased ectoparasitism in the prevalence *L. stenopsis*. For the others ectoparasite (lice and ticks) species, no patterns were observed. The most likely reasons for this result may be related to gender differences in the social structure, phenology and prevalence of sarcoptic mange. Female ibexes are generally more gregarious which should increase the number and duration of body-to-body contacts and therefore the parasite prevalence, abundance and spread [58]. Almost one-third of females were sampled during the periparturient period. At this time, females are particularly susceptible to infestations due to immunosuppression. Further, the prevalence and severity of mange is significantly higher in males than in females [59] which may influence the burdens of ectoparasites (lice and ticks) among host sexes. The immune response of Iberian ibexes to *S. scabiei* is not influenced by host age [60] therefore, this variable was not included in the analysis. Additionally, our data also does not support that differences in vulnerability to parasitism are related to the age classes considered (yearlings and adults).

Regarding the expected changes that *S. scabiei* infestation may promote in ectoparasite community structure and composition, we reported differences in species co-occurrence only at early stages of *S. scabiei* infestation. This fact may indicate that infracommunities are resistant and resilient to external perturbation because after an early change of ectoparasite communities in response to *S. scabiei* infestation we detected a quick return to the initial condition, i.e. we recorded no significant differences in parasite co-occurrence patterns between healthy and severely infested ibexes. The stability of within-host communities in wild mammals was already experimentally demonstrated for endoparasites [6]. Knowles et al. [6], by reducing the burdens of nematode infestation through anthelminthic administration, reported a surprisingly stability of non-target parasite communities to perturbation. Here, we observed a similar response of ectoparasite communities when exposed to a natural and external perturbation such as the infestation by a highly contagious species. In addition, we recorded that scabietic ibexes reached higher parasite richness faster than healthy ibexes, i.e. the asymptote of species accumulation curves was reached first in scabietic ibexes. We conclude that *S. scabiei* may influence the diversity of infracommunities through alteration of host energy needs, thermoregulation and spatial behaviour. *Sarcoptes scabiei* infestation causes an increase of temperature in the affected skin due to hyperemia which makes infested hosts more prone to detection by ectoparasites. Note that one way of tick host-finding is by sensing heat loss from hosts’ body. Further, the disruption of homeostasis by *S. scabiei* made the movement of ibexes increasingly difficult, i.e. infested ibexes tend to exhibit abnormal movements and to move less than healthy ones [14]. Consequently, the home range of infested ibexes tends to decrease and this may create more chances for body-to-body contact and, therefore, for parasite transmission and spread among hosts [61]. Additionally, scabietic ibexes with lower home ranges use specific areas more intensively; this poses an increased risk of new infestations and several opportunities for some ectoparasites to attach. Using radio-collars it may be possible to confirm how the parasite diversity varies in relation to host home range and to which extent the severity of *S. scabiei* infestation affects the spatial behaviour of infested hosts.

## Conclusions

Our study broadens our understanding of the dynamics of infracommunities and constitutes the first insight into the mechanisms underpinning co-infestation in a wild Iberian host experiencing a highly contagious mite infestation. More precisely, we explored how ecosystem engineering and allogenic processes carried out by a highly contagious parasite may impact the features of ectoparasite community. In our study, the numerical response of ectoparasite communities to external perturbations was highly variable and our results suggested that the presence of one highly contagious parasite may not negatively influence the presence and abundance of lice and ticks. Future refinement of sample collection aiming to control for uneven sampling effort and the potential bias caused by host and parasite phenology would serve to increase the reliability of our results and to assess the role of climatic fluctuations on the critical stages of tick’s development. The incorporation of further drivers, either ecologically related variables such as the habitat structure and altitude or epidemiologically related variables such as host population density and individual home range, will allow to make better predictions about the influence of external factors on both parasite counts and community structure. The quantification of attachment sites should be included in future studies to test the spatial competition between ectoparasite species within host. We advocate that manipulative experiments need to be run in order to clarify cause-effect relationships. Such detailed information will allow us to use advanced analytical tools (e.g. mechanistic models) to analyse, in greater detail, the mechanisms of parasite interactions and competitive exclusion at host level.

## Abbreviations

CART, Classification and Regression Trees; CI, Confidence Interval; NDVI, Normalized Difference Vegetation Index; MODIS, Moderate-Resolution Imaging Spectroradiometer; SNNS, Sierra Nevada Natural Space

## References

[1] Rynkiewicz EC, Pedersen AB, Fenton A (2015). An ecosystem approach to understanding and managing within-host parasite community dynamics. Trends Parasitol.

[2] Mideo N (2009). Parasite adaptations to within-host competition. Trends Parasitol.

[3] Johnson PTJ, de Roode JC, Fenton A. Why infectious disease research needs community ecology. Science 2015;349:1259504. doi:10.1126/science.1259504. 10.1126/science.1259504 PMC4863701 26339035

[4] Telfer S, Lambin X, Birtles R, Beldomenico P, Burthe S, Paterson S, Begon M (2010). Species interactions in a parasite community drive infection risk in a wildlife population. Science.

[5] Bandilla M, Valtonen ET, Suomalainen L-R, Aphalo PJ, Hakalahti T (2006). A link between ectoparasite infection and susceptibility to bacterial disease in rainbow trout. Int J Parasitol.

[6] Knowles SCL, Fenton A, Petchey OL, Jones TR, Barber R, Pedersen AB (2013). Stability of within-host parasite communities in a wild mammal system. Proc R Soc B.

[7] Pedersen AB, Fenton A (2007). Emphasizing the ecology in parasite community ecology. Trends Ecol Evol.

[8] Pedersen AB, Antonovics J (2013). Anthelmintic treatment alters the parasite community in a wild mouse host. Biol Lett.

[9] Lutermann H, Fagir DM, Bennett NC (2015). Complex interactions within the ectoparasite community of the eastern rock sengi (*Elephantulus myurus*). Int J Parasitol Parasites Wildl.

[10] Hoffmann S, Horak IG, Bennett NC, Lutermann H (2016). Evidence for interspecific interactions in the ectoparasite infracommunity of a wild mammal. Parasit Vectors.

[11] Thomas F, Poulin R, Meeüs T, Guégan J-F, Renaud F (1999). Parasites and ecosystem engineering: what roles could they play?. Oikos.

[12] Labaude S, Rigaud T, Cézilly F (2015). Host manipulation in the face of environmental changes: Ecological consequences. Int J Parasitol Parasites Wildl.

[13] Turchetto S, Obber F, Permunian R, Vendrami S, Lorenzetto M, Ferré N (2014). Spatial and temporal explorative analysis of sarcoptic mange in Alpine chamois (*Rupicapra r. rupicapra*). Hystrix.

[14] Pérez JM, Ruiz-Martinez I, Granados JE, Soriguer RC, Fandos P (1997). The dynamics of sarcoptic mange in the ibex population of Sierra Nevada in Spain - Influence of climatic factors. J Wild Res.

[15] León-Vizcaíno L, de Ybáñez MR R, Cubero MJ, Ortíz JM, Espinosa J, Pérez L (1999). Sarcoptic mange in Spanish ibex from Spain. J Wildl Dis.

[16] Pence DB, Ueckerman E (2002). Sarcoptic mange in wildlife. Rev Sci Tech Off Int Epiz.

[17] Wall R, Shearer D (2001). Veterinary Ectoparasites. Biology, Pathology & Control. 2nd ed.

[18] Ellse L, Burden FA, Wall R (2014). Seasonal infestation of donkeys by lice: Phenology, risk factors and management. Vet Parasitol.

[19] Estrada-Peña A, de la Fuente J (2014). The ecology of ticks and epidemiology of tick-borne viral diseases. Antiviral Res.

[20] Krasnov BR, Bordes F, Khokhlova IS, Morand S (2012). Gender-biased parasitism in small mammals: patterns, mechanisms, consequences. Mammalia.

[21] Kiffner C, Stanko M, Morand S, Khokhlova IS, Shenbrot GI, Laudisoit A, et al. Variable effects of host characteristics on species richness of flea infracommunities in rodents from three continents. Parasitol Res. 2014;113:2777–88. 10.1007/s00436-014-3937-2 24820040

[22] Navarro-Gonzalez N, Verheyden H, Hoste H, Cargnelutti B, Lourtet B, Merlet J (2011). Diet quality and immunocompetence influence parasite load of roe deer in a fragmented landscape. Eur J Wildlife Res.

[23] Boyer N, Réale D, Marmet J, Pisanu B, Chapuis J-L (2010). Personality, space use and tick load in an introduced population of Siberian chipmunks *Tamias sibiricus*. J Anim Ecol.

[24] Moore SL, Wilson K (2002). Parasites as a viability cost of sexual selection in natural populations of mammals. Science.

[25] Granados JE, Pérez JE, Márquez FJ, Serrano E, Soriguer RC, Fandos P (2001). La cabra montés (*Capra pyrenaica*, Schinz 1838). Galemys.

[26] Poulin R (2001). Interactions between species and the structure of helminth communities. Parasitology.

[27] Pérez JM, Granados JE, Soriguer RC, Fandos P, Márquez FJ, Crampe JP (2002). Distribution, status and conservation problems of the Spanish Ibex, *Capra pyrenaica* (Mammalia: Artiodactyla). Mammal Rev.

[28] Kottek M, Grieser J, Beck C, Rudolf B, Rubel F (2006). World Map of the Köppen-Geiger climate classification updated. Meteorol Z.

[29] Pérez-Luque AJ, Bonet FJ, Pérez-Pérez R, Aspizua R, Lorite J, Zamora R (2014). Sinfonevada: Dataset of floristic diversity in Sierra Nevada forests (SE Spain). PhytoKeys.

[30] Fandos P (1991). La cabra montés *Capra pyrenaica* en el Parque Natural de las Sierras de Cazorla, Segura y Las Villas.

[31] Pérez JM, Granados JE, Sarasa M, Serrano E (2011). Usefulness of estimated surface area of damaged skin as a proxy of mite load in the monitoring of sarcoptic mange in free-ranging populations of Iberian wild goat, *Capra pyrenaica*. Vet Parasitol..

[32] Rodríguez F, Jiménez A, Martín-Mateo MP (1980). Primeras citas de malófagos parásitos de *Capra pyrenaica hispanica*. Nouv Rev Entomol.

[33] Pajot FX (2000). Les poux (Insecta, Anoplura) de la région afrotropicale.

[34] Habela M, Peña J, Corchero E, Sevilla RG (2000). Garrapatas y hemoparásitos transmitidos de interes veterinario en España. Manual práctico para su identificación. 1st ed.

[35] Calvete C, Estrada R, Lucientes J, Estrada A (2003). Ectoparasite ticks and chewing lice of red-legged partridge, *Alectoris rufa*, in Spain. Med Vet Entomol.

[36] Pettorelli N (2013). The Normalized Difference Vegetation Index.

[37] 37.Gu Y, Hunt E, Wardlow B, Basara JB, Brown JF, Verdin JP. Evaluation of MODIS NDVI and NDWI for vegetation drought monitoring using Oklahoma Mesonet soil moisture data. Geophys Res Lett. 2008;35. doi:10.1029/2008GL035772.

[38] Carvalho J, Granados JE, López Olvera JR, Pérez JM, Fandos P, Soriguer RC, Velarde R, Fonseca C, Pettorelli N, Serrano E (2015). Sarcoptic mange breaks up bottom-up regulation of body condition in a large herbivore population. Parasit Vectors.

[39] Zar JH (1999). Biostatistical analysis.

[40] Bush AO, Lafferty KD, Lotz JM, Shostak AW (1997). Parasitology meets ecology on its own terms: Margolis et al. revisited. J Parasitol.

[41] Reiczigel J, Lang Z, Rózsa L, Tóthmérész B (2005). Properties of crowding indices and statistical tools to analyze crowding data. J Parasitol.

[42] Reiczigel J (2003). Confidence intervals for the binomial parameter: some new considerations. Stat Med.

[43] Breiman L, Friedman JH, Olshen RA, Stone CJ (1984). Classification and regression trees.

[44] Williams G (2011). Data Mining with Rattle and R. The Art of Excavating Data for Knowledge Discovery. 1st ed.

[45] Therneau T, Atkinson B, Ripley B. Package “rpart”: Recursive partitioning and regression trees. 2013. http://cran.r-project.org/web/packages/rpart/index.html. Accessed 10 Nov 2015.

[46] Milborrow S. Package “rpart.plot”: Plot rpart Models. An Enhanced Version of plot.rpart. 2012. http://cran.r-project.org/web/packages/rpart.plot/index.html. Accessed 12 Nov 2015.

[47] R Core Team. R: A language and environment for statistical computing. R Foundation for Statistical Computing. 2014. http://www.R-project.org/. Accessed 30 Oct 2015.

[48] Diamond JM, Cody ML, Diamond JM (1995). Assembly of species communities. Ecology and evolution of communities.

[49] Stone L, Roberts A (1990). The checkerboard score and species distributions. Oecologia.

[50] Dove ADM, Cribb TH (2006). Species accumulation curves and their applications in parasite ecology. Trends Parasitol.

[51] Gotelli NJ, Entsminger GL. Ecosim: Null Models Software for Ecology, Version 7.72. 2001; Acquired Intelligence Inc, & Kesey-Bear.

[52] MacArthur R, Levins R (1967). The limiting similarity, convergence, and divergence of coexisting species. Am Nat.

[53] Samuel WM, Pybus MJ, Kocan AA (2001). Parasitic Diseases of Wild Mammals.

[54] James PJ, Moon RD, Brown DR (1998). Seasonal dynamics and variation among sheep in densities of the sheep biting louse. Bovicola Ovis Int J Parasitol.

[55] Gawler R, Coles GC, Stafford KA (2005). Prevalence and distribution of the horse louse, *Werneckiella equi equi*, on hides collected at a horse abattoir in south-west England. Vet Rec.

[56] Noori NV, Rahbari S, Bokaei S (2012). The seasonal activity of *Rhipicephalus bursa* in Cattle in Amol (Northern Iran). World Appl Sci J.

[57] Papazahaiadou MG, Papadopoulos EG, Himonas CA (1995). Seasonal activity of ixodid ticks on goats in northern Greece. Vet Rec.

[58] Altizer S, Nunn CL, Thrall PH, Gittleman JL, Antonovics J, Cunningham AA (2003). Social organization and parasite risk in mammals: Integrating theory and empirical studies. Annu Rev Ecol Evol Syst.

[59] López-Olvera JR, Serrano E, Armenteros A, Pérez JM, Fandos P, Carvalho J (2015). Sex-biased severity of sarcoptic mange at the same biological cost in a sexually dimorphic ungulate. Parasit Vectors.

[60] Sarasa M, Rambozzi L, Rossi L, Meneguz PG, Serrano E, Granados J-E (2010). *Sarcoptes scabiei*: Specific immune response to sarcoptic mange in the Iberian ibex *Capra pyrenaica* depends on previous exposure and sex. Exp Parasitol.

[61] Bordes F, Morand S, Kelt DA, Van Vuren DH (2009). Home range and parasite diversity in mammals. Am Nat.

[62] Jones CG, Lawton JH, Shachak M (1994). Organisms as ecosystem engineers. Oikos.

[63] Alizon S, Hurford A, Mideo N, Baalen V (2009). Virulence evolution and the trade-off hypothesis: history, current state of affairs and the future. J Evol Biol.

[64] Casadevall A, Pirofski L (2001). Host-pathogen interactions: the attributes of virulence. J Infect Dis.

